# P-187. Clinical Profile of Atypical Manifestations of Dengue Fever and Severe dengue fever in children in Argentina

**DOI:** 10.1093/ofid/ofae631.391

**Published:** 2025-01-29

**Authors:** Ximena S Juarez, Laura Beatriz Miño, Eugenia Sevilla, Valeria Aprea, Maria Fernanda Gonzalez, Vivian Bokser, Emilce Haleblian, maria Jose Rial

**Affiliations:** Hospital de Niños P. de Elizalde, Buenos Aires, Buenos Aires, Argentina; Hospital General Niños Pedro de Elizalde, buenos aires, Ciudad Autonoma de Buenos Aires, Argentina; Hospital General Niños Pedro de Elizalde, buenos aires, Ciudad Autonoma de Buenos Aires, Argentina; Hospital General Niños Pedro de Elizalde, buenos aires, Ciudad Autonoma de Buenos Aires, Argentina; Hospital General Niños Pedro de Elizalde, buenos aires, Ciudad Autonoma de Buenos Aires, Argentina; Hospital General Niños Pedro de Elizalde, buenos aires, Ciudad Autonoma de Buenos Aires, Argentina; Hospital General Niños Pedro de Elizalde, buenos aires, Ciudad Autonoma de Buenos Aires, Argentina; Hospital General Niños Pedro de Elizalde, buenos aires, Ciudad Autonoma de Buenos Aires, Argentina

## Abstract

**Background:**

The clinical presentation of dengue fever has a wide spectrum; unussual manifestations have been reported and with the rising disease burden, atypical manifestations are also on rise, but are underreported and often missed due to the lack of awareness among health care professionals. In Argentina we are witnessing the largest historical outbreak of dengue in 2024.

The objective of the study is to study the clinical profile and outcome of atypical and severe manifestations of dengue fever in children.

**Methods:**

All children (0–18 y of age) confirmed as dengue fever at a tertiary care hospital at Buenos Aires, Argentina between January 2023-April 2024 were reviewed retrospectively from hospital case records. The diagnosis was confirmed by NS1 antigen/IgM based ELISA or inmunocromatography test, PCR. The data was analyzed using EpiInfo 7.2

**Results:**

Out of **213** children admitted with dengue fever, 7 % had severe dengue. Atypical manifestations were seen in 37 cases ( 17 %). Mean age of presentation was 6.5 (5.2) y. M: F ratio was 1.1:1. The common manifestations of severe dengue infection were shock (80 %), bleeding (20 %) and multi organ dysfunction (6 %).

The most common atypical manifestations of dengue fever were hepatitis (19%), seizures (8 %), acalculous cholecystitis (8%), myositis (8%),pneumonia (8%), febrile infant without focus (8%), febrile diarrhea (19 %). The other atypical manifestations present were ataxia, meningitis, acute respiratory distress syndrome (ARDS), myocarditis, lymphadenitis, prolonged fever, pericardial and pleural effusion. There was one death (0.4 %).

**Conclusion:**

The atypical manifestations of dengue fever are no more a rare entity. Clinicians should have a high index of suspicion and surveillance for atypical manifestations of dengue fever as lack of timely detection and management could be fatal.

**Disclosures:**

**All Authors**: No reported disclosures

